# Simulated patient methodology for the investigation of community pharmacy practice: a worldwide success story

**DOI:** 10.25122/jml-2025-0138

**Published:** 2025-09

**Authors:** Christian Kunow, Bernhard Langer

**Affiliations:** 1 Department of Health, Nursing, Management, Neubrandenburg University of Applied Sciences, Neubrandenburg, Germany

**Keywords:** patient simulation, community pharmacy services, standards, observation

## Abstract

The simulated patient methodology (SPM), a form of participant observation, is generally recommended in the international literature. SPM studies investigating community pharmacy (CP) practice have been conducted in at least 52 countries from all regions of the world, with the number of publications per year increasing. Not only 'traditional' visits, but also calls are used in SPM studies. Accordingly, in addition to reviews of visits, reviews of calls are planned. The interest in the SPM is already so great that not only worldwide reviews, but also reviews on specific regions and individual countries, have been published. Finally, a checklist called CRiSPHe is available to help researchers report their studies using the simulated patient method. It was developed through a Delphi study focused on pharmacy and has since been refined. SPM is now considered the 'gold standard' in the international literature for investigating CP practice.

## To the Editor,

The simulated patient methodology (SPM), which did not originate in health care but was used in the 1940s by private detectives as "mystery shopping" in banks and retail stores to uncover employee theft [1], is a form of participant observation. Simulated patients (SPs) covertly contact a health service and simulate participation in a seemingly real service process based on a pre-defined scenario [2]. The use of SPM is generally recommended in the international literature [2,3]. In addition to health insurance, hospitals, primary care, and community pharmacies (CPs) can be considered as investigation settings. A specific investigation of CP practice is also warranted, as CPs play a significant role in healthcare.

SPM for the investigation of CP practice can now be considered a worldwide success story. SPM studies have been conducted in at least 52 countries across all regions of the world [4,5], with the number of publications per year increasing from an average of 2 (1976 to 2005) [4] to 13 (2006 to 2016) [5] and now to 17 (2018 to 2020) [6]. Not only "traditional" visits, but also calls are used in SPM studies. Accordingly, in addition to reviews of visits [4,5], reviews of calls are planned [7]. The interest in the SPM is already so great that not only worldwide reviews [4,5], but also reviews on selected regions [8] and even on individual countries [9] have been published. Finally, a checklist for reporting research using the simulated patient methodology in Health (CRiSPHe) has been developed through a Delphi study focused on pharmacy and has since been refined [10].

The use of SPM has the advantage of avoiding the social desirability bias typically associated with surveys, interviews, and clinical vignettes, as well as the Hawthorne effect commonly found in non-participant observations. However, intra- and inter-observer variabilities are possible [3]. The global success story of investigating CP practice is underscored by the fact that the SPM is now considered the "gold standard" [11] in the international literature, largely due to its high internal validity [2]. There is also criticism that the SPM can only provide a small picture of everyday pharmacy practice and therefore has limited external validity [2]. However, a specific application of the SPM – in the sense of multiple contacts with a single CP involving different SPs at different times, using differently designed scenarios – can provide the most comprehensive picture of everyday pharmacy practice and increase external validity [11]. Even if this is associated with a reduction in internal validity due to the trade-off situation [2] and requires considerable personnel and organisational effort [11], this has not yet stopped the worldwide success of SPM for the investigation of CP practice.

## References

[1] Wisniewska M, Dahlgaard-Park SM (2015). Mystery shopping. The SAGE encyclopedia of quality and the service economy.

[2] Collins JC, Chong WW, de Almeida Neto AC, Moles RJ, Schneider CR (2021). The simulated patient method: Design and application in health services research. Res Social Adm Pharm.

[3] Caamaño F, Ruano A, Figueiras A, Gestal Otero JJ (2002). Data collection methods for analyzing the quality of the dispensing in pharmacies. Pharm World Sci.

[4] Watson MC, Norris P, Granas AG (2006). A systematic review of the use of simulated patients and pharmacy practice research. Int J Pharm Pract.

[5] Björnsdottir I, Granas AG, Bradley A, Norris P (2020). A systematic review of the use of simulated patient methodology in pharmacy practice research from 2006 to 2016. Int J Pharm Pract.

[6] Amaratunge S, Harrison M, Perry D, Bond C, Ceulemans M, Foulon V (2022). Assessing the reporting quality of simulated patient studies in pharmacy research using a novel checklist (CRiSP). Res Social Adm Pharm.

[7] Kunow C, Langer B (2023). Using the simulated patient methodology in the form of mystery calls in community pharmacy practice research: A scoping review protocol. Pharmacy.

[8] Boura F, Al-Tabakha M, Hassan N, Darwich M (2022). Critical appraisal of simulated patient methodology to assess the practice of community pharmacist in the Middle East and North Africa region: A systematic review. Pharm Pract (Granada).

[9] Kunow C, Langer B (2021). Using the simulated patient methodology to assess the quality of counselling in German community pharmacies: A systematic review from 2005 to 2018. Int J Pharm Pharm Sci.

[10] Park JS, Page A, Clifford R, Bond C, Seubert L (2024). Refining the CRiSPHe (checklist for reporting research using a simulated patient methodology in Health): a Delphi study. Int J Pharm Pract.

[11] Kunow C, Langer B (2024). Simulated patient methodology as a “gold standard” in community pharmacy practice: Response to criticism. World J Methodol.

